# Editorial: Binge drinking in the adolescent and young brain, volume II

**DOI:** 10.3389/fpsyg.2023.1344363

**Published:** 2024-01-10

**Authors:** Eduardo López-Caneda, Séverine Lannoy, Salvatore Campanella, Carina Carbia

**Affiliations:** ^1^Psychological Neuroscience Lab, Psychology Research Center (CIPsi), School of Psychology, University of Minho, Braga, Portugal; ^2^Virginia Institute for Psychiatric and Behavioral Genetics, Virginia Commonwealth University, Richmond, VA, United States; ^3^Laboratoire de Psychologie Médicale et d'Addictologie, ULB Neuroscience Institute (UNI), Centre Hospitalier Universitaire (CHU) Brugmann-Université Libre de Bruxelles (U.L.B.), Brussels, Belgium; ^4^Louvain Experimental Psychopathology Research Group (UCLEP), Psychological Sciences Research Institute (IPSY), UCLouvain, Louvain, Belgium

**Keywords:** alcohol, binge drinking, adolescence, young adulthood, brain, cognitive function

Alcohol is by far the most widely used and consumed psychoactive agent in the world. According to current estimates, there are more than 2.4 million active consumers worldwide (about one third of the world's population; Griswold et al., 2018). Almost all societies that consume alcohol show, in turn, related physical, social, and psychological problems (Babor et al., 2022). In this sense, alcohol misuse is recognized as being responsible for 3 million fatalities every year-around 4% of all deaths globally (World Health Organization, 2018). This percentage markedly increases in the American and European youth, where alcohol is associated with nearly three in 10 deaths in people aged 15–29 years (World Health Organization, 2011).

Adolescence and young adulthood are periods of vulnerability in which many psychiatric disorders such as anxiety, depression or substance abuse manifest for the first time (Paus et al., 2008; Hawkins, 2009). In addition, significant maturational changes take place on the brain during this crucial developmental period, which is considered to last up to 24 years of age (Sawyer et al., 2018). The ongoing neuromaturation seems to involve greater vulnerability–in comparison to adulthood- to disruptive events in the brain such as binge drinking (BD; Spear, 2016; Chung et al., 2018). This pattern of consumption is commonly defined as the consumption of five or more standard drinks (four or more for females) in about 2 h on at least 1 day in the past month (National Institute of Alcohol Abuse and Alcoholism, 2020; Substance Abuse and Mental Health Services Administration, 2022). It constitutes a special concern for this population, as it has been associated with structural and functional impairments in still-maturing regions (e.g., the prefrontal cortex; Cservenka and Brumback, 2017; Lees et al., 2019; Pérez-García et al., 2022) and neuropsychological and neurofunctional deficits in executive functions (e.g., inhibitory control; Carbia et al., 2018; Almeida-Antunes et al., 2021; Seabra et al., 2023), together with a high risk of developing future alcohol addiction (Crews et al., 2016; Ventura-Cots et al., 2017; Tavolacci et al., 2019).

Collectively, the high prevalence of BD at this age along with its serious health problems has led to a significant increase in the number of investigations of this phenomenon, reaching a 7-fold increase between 2000 and 2018, with a slight decrease in the last few years (see Figure 1). Due to the growing interest concerning this pattern of heavy alcohol drinking, in 2019 we published the first volume of the Research Topic “*Binge drinking in the adolescent and young brain*” (López-Caneda et al., 2019). As showed in this issue, the evidence gathered from more than 20 published papers points to brain anomalies associated with BD at different levels: cellular (e.g., Nickell et al., 2017), structural (e.g., Sousa et al., 2017), functional (e.g., Folgueira-Ares et al., 2017), and cognitive (e.g., Gil-Hernández et al., 2017). In this second volume, we aim to update the latest research related to BD in order to bring the most recent findings that shed new light on this complex and challenging problem in alcohol research and society as a whole.

**Figure 1:**
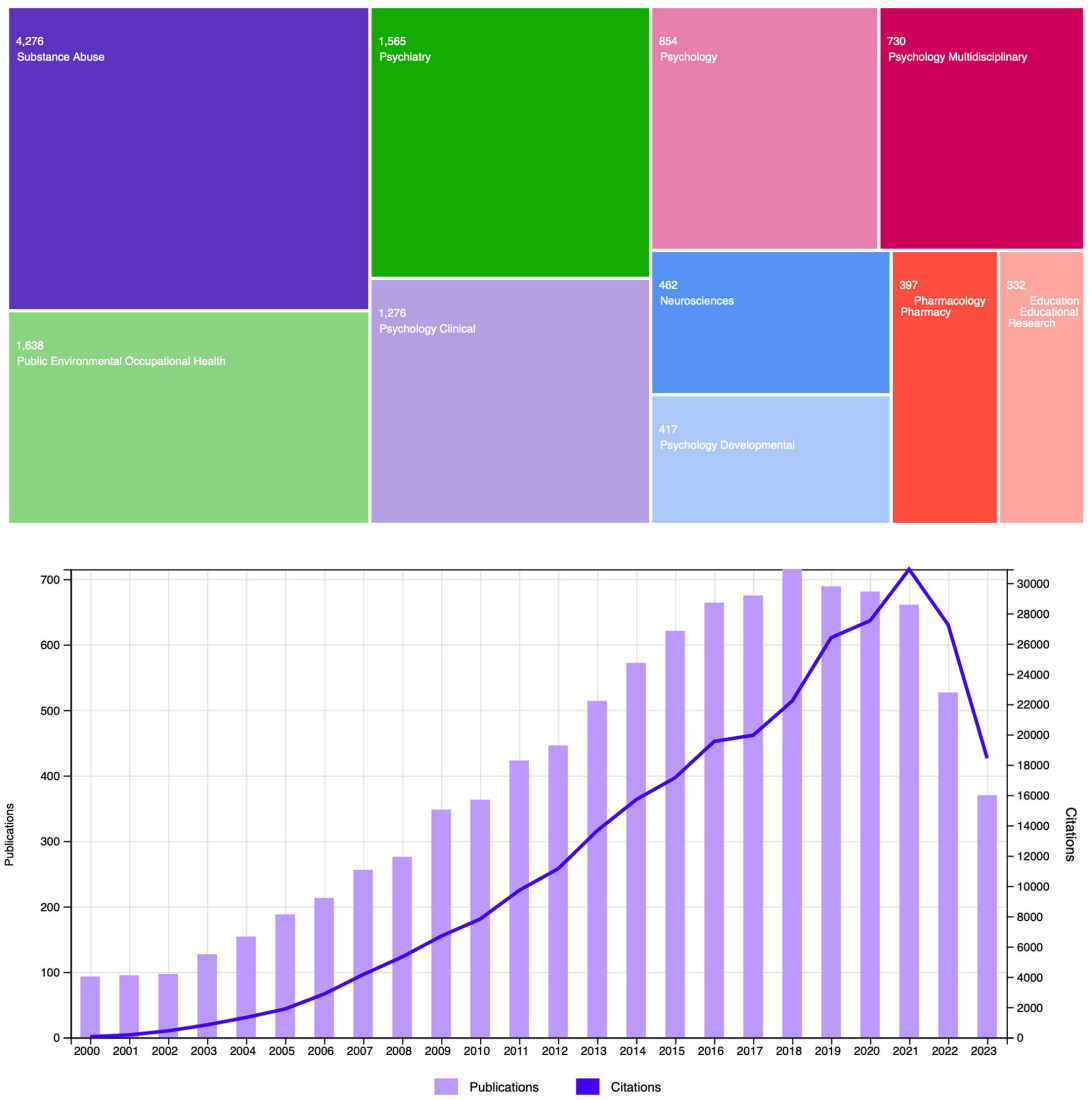
Number of articles related to binge drinking during adolescence and youth for the period January 2000–November 2023, per research area **(above)** and per year-including the number of articles citing research in this topic **(below)**. The search strategy was conducted in Web of Science with the following key terms: ((“binge drinking” OR “binge drinkers” OR “heavy drinking” OR “heavy drinkers” OR “heavy episodic drinking” OR “college drinking” OR “college drinkers” OR “social drinkers”) AND (adolescen* OR youth* OR teen* OR “young” OR “young adults” OR “college students” OR “university students”)).

Firstly, with regard to preclinical studies, three articles included in this Research Topic employed animal models to explore the potential neurotoxic effects of BD. Specifically, the study by Sauton et al. longitudinally assessed decision-making capacities, memory and anxiety-like behavior as well as striatal dopaminergic signaling in male and female rats with a history of BD exposure. Voluntary BD intake was associated with weak decision-making abilities in males, impaired dopamine transmission in the core of the nucleus accumbens (NAcc) in females and increased motor impulsivity in both sexes, suggesting that chronic voluntary BD exposure may lead to a vicious cycle resulting in BD perpetuation and contributing to alcohol dependence vulnerability. In the same line, Jeanblanc et al. assessed the effect of different Baclofen (a GABAB receptor agonist used to treat alcohol use disorder) compounds on daily BD behavior and on dopamine release in the core of the NAcc in rats. Findings showed that both RS(±)-Baclofen and R(+)-Baclofen enantiomers were effective in reducing alcohol intake in animals with a BD-type pattern of alcohol self-administration, particularly in male rats, and that both forms of Baclofen decreased dopamine release in the NAcc in control animals-potentially reducing the rewarding properties of alcohol-, an effect that was lost in the BD group. The third preclinical study, conducted by Jimenez Chavez et al., concluded that BD history in adult and adolescent mice induces relatively few signs of alcohol withdrawal-related negative affect, although several design constraints were raised by the authors as potential causes of this unexpected outcome (e.g., space employed for behavioral testing, locations of the colony rooms in which mice consumed alcohol, small sample size for the replication study). Despite these limitations, authors observed that a 2-week BD history was sufficient to induce some signs of mild cognitive impairment -namely, impaired working and spatial memory-, particularly in adolescent-onset binge drinkers (BDs), which persist after 1 month following the cessation of drinking.

Several human studies encompassed in this Research Topic have also reported abnormal memory functioning associated with a BD pattern. Accordingly, Vinader-Caerols and Monleón described impair faces memory -assessed by the Wechsler Memory Scale (WMS-III; Wechsler, 2004) in male adolescent BDs in comparison with aged-matched abstainers and BDs with cannabis consumption. After receiving a risk dose of alcohol, female BDs performed better than abstainers in the scene memory test of the WMS-III, indicating a possible cognitive tolerance to acute alcohol intake in woman. Similarly, findings from Kang and Kim's study revealed that college students who engage in BD display difficulties with verbal and non-verbal memory-showing lower performance in the free recall condition of the California Verbal Learning Test and in the delayed recall condition of the Rey-Osterrieth Complex Figure Test-as well as deficits in cognitive control-as indicated by the lower number of categories completed in the Wisconsin Card Sorting Test. In another study assessing memory, Johnstone et al. investigated the negative emotional memory bias in the context of BD and problematic alcohol use and its potential association with coping motivations and depressive symptoms. Results support that engaging in BD as a coping mechanism for negative affect or experiencing elevated depression was indirectly related to more negative (self-referent) memory biases, particularly in females. In a related vein, in the study by Cortés-Tomás et al., coping with depressive moods also emerged as a significant factor in explaining risk to engage in BD in young females. In addition, positive expectancies toward alcohol and social and enhancement motives were also important determinants of the BD behavior.

In addition, two electrophysiological studies reported abnormal neural activity linked to memory processes in young with a BD pattern. Rodríguez Holguín et al. analyzed verbal memory during a verbal paired associates learning task using electroencephalography (EEG) and findings revealed that the old/new effect -i.e., larger amplitudes in event-related potentials for old (i.e., previously studied) items in comparison with new items- was absent in the BD relatively to the control group, indicating anomalous brain activity during the recognition of previously learned words in young BDs. In addition, BDs also displayed poorer recall of previously studied words in a version of the California Verbal Learning Test, which is consistent with a verbal memory impairment in this population. These results are congruent with the EEG study conducted by Huang et al., which found an absence of the subsequent memory effect in BDs as compared to aged-matched light drinkers. Indeed, BDs showed attenuated (instead of increased) event-related theta activity and lack of theta phase-locking (an index of neural synchrony) between frontal and posterior/temporal regions during encoding of images that were prospectively retained over 6 months in remote memory, suggesting BD-induced disturbances in brain areas critical for memory formation. In a 2-year longitudinal study, Antón-Toro et al. recorded the brain electrophysiological activity of BDs and their control peers during an inhibitory control (Go/No-Go) task by magnetoencephalography (MEG) in two stages: the first one prior to the onset of alcohol consumption and, subsequently, 2 years later when some of them had started a BD-type pattern. Results showed that, before alcohol use initiation, increased functional connectivity (in the beta band) in prefrontal and temporal regions was associated with augmented risk of developing BD 2 years later. Furthermore, adolescents who transitioned into BD displayed a marked decline of functional synchronization in the prefrontal, temporal and parietal cortices along the follow-up period which, according to the authors, was suggestive of a disruption in the typical neurodevelopmental trajectory for this age. The study protocol by Almeida-Antunes et al. also proposed to analyze the functional connectivity patterns linked to inhibitory control processes, but in this case they intended to explore the memory inhibition/suppression abilities in BDs and non-/light-drinkers in two occasions, before and after several sessions of memory inhibition training (involving cognitive and electrical stimulation). With this aim, authors expect to be able to determine by the first time potential neurofunctional anomalies in the memory inhibition processes linked to BD habits and, subsequently, improve the abilities to suppress alcohol-related memories, which might have a significant impact in alcohol use and craving.

The only longitudinal study examining brain structural changes associated with BD was conducted by Pérez-García et al. Findings revealed that a continued BD pattern in emerging adults is linked to gray matter anomalies in regions related to reward processing (reduced volume in the right NAcc in male BDs), emotional regulation (larger surface area in the left insula in both male and female BDs) and executive functions (thinner cortices in the right rostral middle frontal gyrus in male BDs). Similarly, a critical review conducted by Tetteh-Quarshie and Risher emphasizes the factors that lead to BD-induced brain abnormalities, highlighting the role of the maturational changes occurring during adolescence as a key factor when explaining the neurocognitive and neurostructural effects of BD. Also focusing on the still-maturing brain, the opinion article by Angioletti and Balconi introduces the relationship between BD and problem gambling in adolescents, indicating a connection between the two behaviors. The article underscores the maturational imbalance between the fully mature, over activated reward system and the still-maturing executive system as a potential vehicle toward risky behaviors-including BD and problem gambling- and also underlines the need for tailored preventive and treatment interventions for adolescents with BD and risky gambling, focusing on cognitive factors, especially executive functions and decision-making.

Finally, an objective and homogeneous definition/characterization of BD seems crucial to enable comparisons of findings across different studies. To this end, André et al. developed a new tool to identify and classify BD patterns, applicable to both genders. Authors introduced a 5-item model-based on the Alcohol Use Disorders Identification Test (Saunders et al., 1993) and the Alcohol Use Questionnaire (Townshend and Duka, 2002)-combining multiple key factors of BD, such as quantity/frequency of consumption (6-drink frequency), behavior (speed of consumption), and physiology (frequency of drunkenness and hangover), resulting in a comprehensive and valuable measure for assessing BD severity and categorizing drinking patterns across diverse populations.

In summary, the collection of articles included in this second volume covers a range of important topics related to BD during adolescence and young adulthood, including the behavioral, emotional, cognitive and neurobiological impact of this heavy pattern of alcohol use. However, further research is still needed in order to better characterize the effects of BD on this population. Particularly, conducting longitudinal studies initiating before the transition from non-/low-drinking into BD will allow for a more accurate assessment of the neurodevelopmental impact and potential causal relationships between BD and brain impairments (Gilpin et al., 2012; Antón-Toro et al., 2021). Likewise, examining the spectrum of alcohol consumption, from light to high-intensity drinkers and individuals with alcohol abuse, would provide a more thorough understanding of how varying levels and patterns of alcohol intake may contribute to different behavioral, emotional, and neurocognitive anomalies (Patrick and Azar, 2018; Maurage et al., 2020). Also, future studies should include a more diverse cross-section of the general population (beyond college students) to increase the applicability and generalization of research outcomes. Additionally, research involving participants with comorbidities frequently associated with heavy alcohol drinking, such as anxiety, depression, or other substance abuse, despite its intrinsic limitations, might shed light on the possible interactions/aggravations of these conditions. In summary, bridging the gaps in the existing literature and integrating it with the existing knowledge can contribute to develop more effective prevention and intervention strategies. Ultimately, this will hopefully help to mitigate some of the detrimental effects of BD on the adolescent and young brain.

## Author contributions

EL-C: Conceptualization, Data curation, Funding acquisition, Writing – original draft. SL: Supervision, Writing – review & editing. SC: Supervision, Writing – review & editing. CC: Supervision, Writing – review & editing.
